# Perivascular epithelioid cell tumor outgrowth from the liver

**DOI:** 10.1016/j.ijscr.2018.10.046

**Published:** 2018-11-10

**Authors:** Mahir Kirnap, Gonca Ozgun, Gokhan Moray, Mehmet Haberal

**Affiliations:** a Departments of Transplantation, Baskent University, Ankara, Turkey; b Departments of Pathology, Baskent University, Ankara, Turkey

**Keywords:** Liver, PEComa, Surgery, Mesenchymal neoplasia

## Abstract

**Introduction:**

Perivascular epithelioid celltumor (PEComa) is a rare mesenchymal neoplasia and can be found in various body sites. On the other hand, hepatic PEComa is very rare, with only a few studies having reported hepatic malignant PEComa. There is no gold standard regarding the use of diagnostic imaging studies. The diagnosis of hepatic PEComa is made by a positive immunohistochemical staining for HMB45 and Melan A. Herein, we discussed the therapeutic and follow-up process of a symptomatic hepatic PEComa case.

**Presentation of case:**

A 22-year-old woman presented with a palpable mass in abdomen. A computerized tomographic examination showed a giant hepatic mass of left lobe origin, which was excised surgically. The pathology result was reported as a PEComa.

**Discussion:**

The diagnostic approach, treatment modalities, and follow-up procedures are not standard. The main treatment modality for PEComa is surgical excision with adequate surgical margin.

**Conclusion:**

A longer follow-up is required for patients with hepatic PEComa because the nature of the disease is not entirely clear.

## Highlights

• Perivascular epithelioid cell tumor is a rare mesenchymal neoplasia and can be found in various body sites.
• The diagnosis of hepatic PEComa is made by a positive immunohistochemical staining for HMB45 and Melan.
• The diagnostic approach, treatment modalities, and follow-up procedures are not standard.
• The main treatment modality for PEComa is surgical excision with adequate surgical margin.

## 1 Introduction

Bonetti et al first defined perivascular epithelioid cells in 1992. The term perivascular epithelioid cell tumor (PEComa) was registered by Zamboni et al in 1996 [1,2]. The World Health Organization defined PEComa as unusual mesenchymal tumors composed of histologically and immunohistochemically distinct perivascular epithelioid cells. PEComa tumor family consists of classical epithelioid angiomyolipoma (AML), lymphangioleiomyomatisis, pulmonary and extrapulmonary tumors [3,4]. There exist several hypotheses for the origin of PEComa. The first one states that PEComa develops from undifferentiated neural crest cells that are capable of synthesizing the phenotype of smooth muscle and melanocytes.

PEComa has been shown to involve many body sites including mediastinum, nasopharyngeal cavity, buccal mucosa, abdominal wall, skin, spinal cord, duodenum, ileum, jejunum, colon, rectum, ligamentum teres and falciform ligament, bile duct, pancreas, urinary bladder, prostate, penis, breast, uterus, cervix, vulva, ovaries, heart, lung, kidneys, base of skull, urinary bladder, and pelvic wall [5]. Hepatic PEComa is very rare. Only a few studies have reported hepatic benign or malignant PEComa [4].

No gold standard exists for diagnostic imaging studies. Hepatic PEComa is diagnosed with a positive immunohistochemical staining with HMB45 and Melan A [6]. Herein, we discussed the therapeutic and follow-up process of a 22-year-old woman who presented with symptomatic, giant hepatic PEComa that radiologically outgrew exophytically from the liver. This work has been reported in line with the SCARE criteria [7].

## 2 Case report

**Fig. 1:**
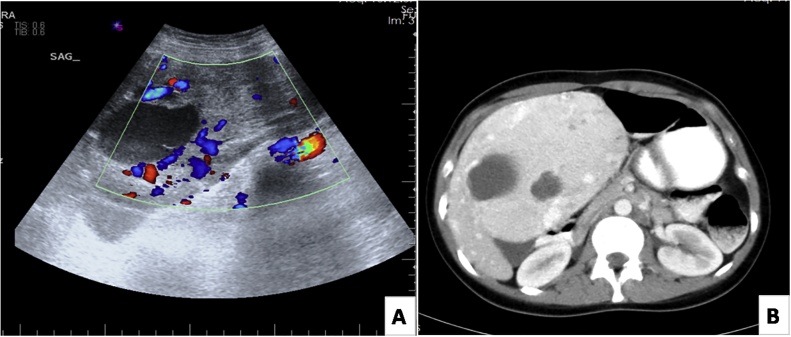
**(A)** Doppler ultrasonogram showing hyperechogenic, cystic, and hypervascular hepatic perivascular epithelioid cell tumor. **(B)** Computed tomography scan of tumor.

A 22-year old woman presented to our clinic with a palpable mass for 6 months. The mass was painless. Her medical history was not remarkable for any disorder. On physical examination she had a palpable mass filling the left upper quadrant and epigastrium. On laboratory examination she had normal levels of total protein, albumin, globulin, alanine aminotransferase, aspartate aminotransferase, blood urea nitrogen, serum creatinine, carbohydrate antigen 19–9 (Ca19-9), carcinoembryonic antigen (CEA) and alpha-fetoprotein (AFP). She also had negative serology for hepatitis B and C viruses. On ultrasonography there was a hypoechoic, solid mass with sharp contours and heterogenous pattern which had a size of 16 x 10 cm and diffuse cystic-degenerative areas and which appears hypervascular on Doppler USG (Fig. 1A). The described mass was considered to reside exophytically in the left lobe of the liver. An urgent abdominal tomography showed a giant solid mass that originated from the inferior part of the medial segment of the left lobe of liver and that extended inferiorly. Its size was approximately 17 × 15 x 11 cm. It had smooth contours and marked hypervascularity. It contained diffuse cystic-degenerative areas. A giant hepatic adenoma was primarily considered in the differential diagnosis, which also included liver tumors of mesenchymal origin or hepatocellular carcinoma on a non-cirrhotic basis (Fig. 1B).

**Fig. 2:**
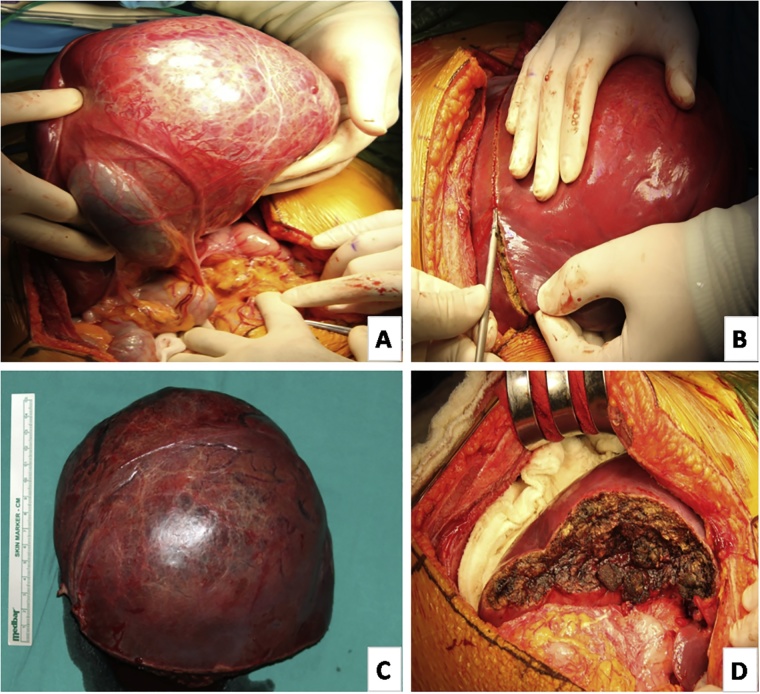
**(A**) Intraoperative view of tumor mass. **(B)** View of surgical margin. **(C)** Size of tumor mass. **(D)** Postoperative cross-sectional area of the liver.

The patient’s abdominal cavity was explored with a subcostal incision. There was a mass with smooth contours, measuring 15 x 12 cm in the left lobe of the liver, which grew exophytically. Other parts of the liver were normal. The mass’s portion out of the liver was of hypervascular appearance that compressed adjacent tissues but was easily separable from them. The mass was excised with liver tissue and gall bladder, with a negative surgical margin, with the help of an ultrasonic dissector and cautery. There was no additional lesion in the abdominal cavity (Fig. 2).

**Fig. 3:**
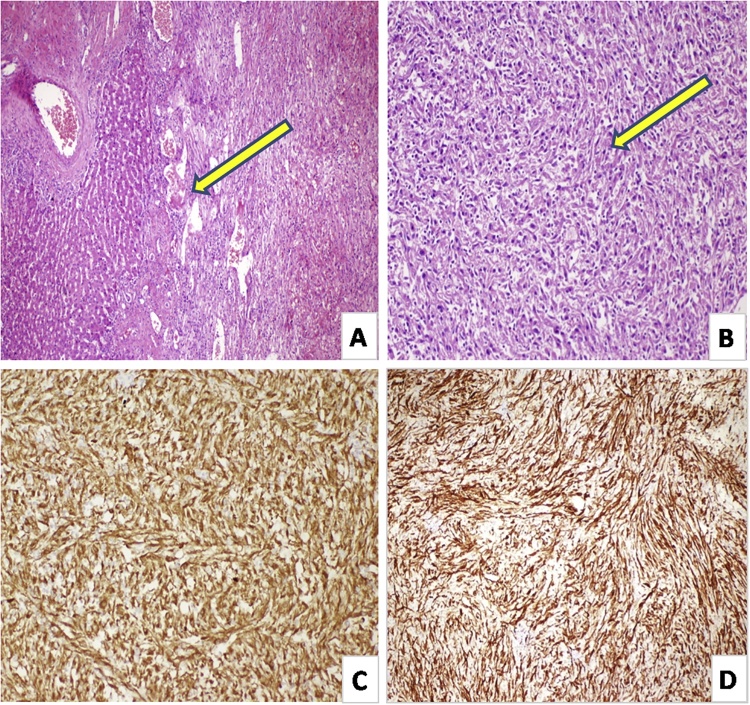
**(A)** Tumor areas infiltrating the hepatic parenchyma (shown with yellow arrow). **(B)** Perivascular epithelioid cells with round to ovoid nuclei and abundant eosinophilic cytoplasm, which are occasionally spindle-shaped but mostly epithelioid (shown with yellow arrow). **(C)** Diffuse positive immunohistochemical staining in cytoplasmic area for HMB-45. **(D)** Diffuse positive immunohistochemical staining in cytoplasmic area for smooth muscle actin.

The macroscopic examination of the hepatic resection material revealed a tumoral lesion with a size of 14 × 12 x 13 cm and a cross-sectional color of yellow, which contained diffuse hemorrhagic and necrotic areas, 2 cm apart from the surgical margin. Sections prepared from the tumor showed that it was separated from the adjacent hepatic parenchyma with a clear border but showed infiltration of the parenchyma in a few foci (Fig. 3A). The tumor was highly cellular, the components of which were spindle in shape from place to place and epithelioid in most areas, and they had round-ovoid nuclei and abundant eosinophilic cytoplasm (Fig. 3B). There were interspersed cells that showed nuclear coarsening. Tumor’s background was highly rich in vascularity and there were interspersed free hemorrhagic foci.

Immunohistochemical study showed negative staining with Pan-CK, Hep-Par, CD117. There was diffuse cytoplasmic positivity with HMB-45 (Fig. 3C) and SMA (Fig. 3D). The background rich vascular network was positively stained with CD34, CD31 and Factor 8 while tumor cells were not. Two mitotic figures were noted under 50 gross magnification. Morphological appearance and immunohistochemical study results suggested a PEComa. Although the criteria for malignancy have not been clearly defined for hepatic PEComas, considering a tumor size greater than 5 cm, presence of more than 1 mitosis under 50 GMA, and infiltrative growth pattern, which have been associated with tumor recurrence or metastatic process for soft tissue or gynecological tumors, the case was considered a malignant PEComa.

**Fig. 4:**
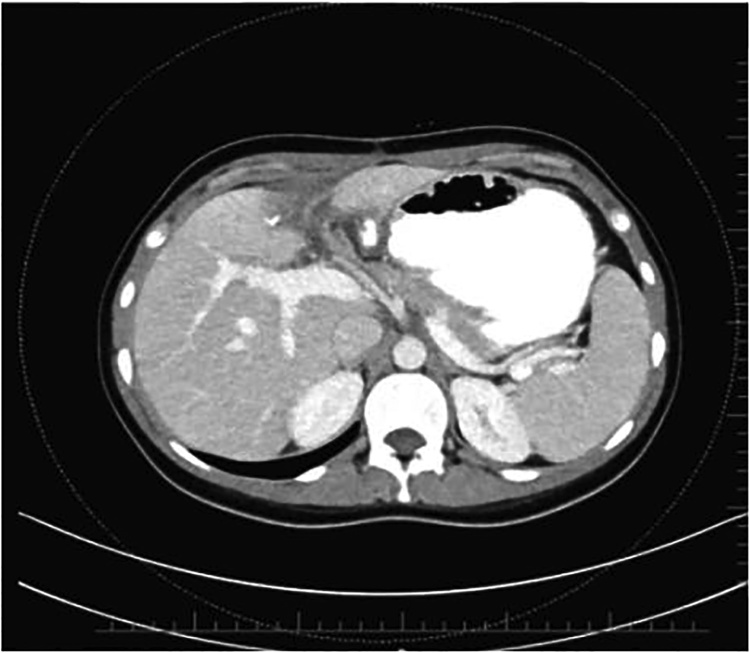
Computed tomographic follow-up image 10 months after surgery.

The patient was discussed in general surgery and oncology councils, which recommend no therapy. The patient recovered uneventfully, and no additional therapy was recommended. She was discharged 3 days after the surgery. She was put under close follow-up; her tri-monthly control tomographic examinations revealed no pathology. She is under follow-up without recurrence 10 months after the surgery (Fig. 4).

## 3 Discussion

Hepatic PEComa cases have been sporadically reported worldwide. The majority of cases are asymptomatic. Hepatic PEComas are most commonly seen between 30 and 50 years of age but they may arise at any age. Some studies have shown that women significantly more commonly develop the disease. Hormones may play an important role in the pathophysiology. However, histogenesis and pathogenesis of the perivascular epithelioid cells are still unclear [8]. Hepatic PEComa produce nonspecific clinical signs and symptoms and they are incidentally detected during physical examination. They produce gastrointestinal symptoms such as abdominal pain, nausea, a dominant palpable mass, and vomiting; the reason of these symptoms increased lesion size causing localized pressure effect or hepatic capsular distention [9]. Our patient was a 22-year-old woman presenting with abdominal swelling, in addition to which there existed no specific clinical symptoms or serological abnormalities.

Clinically, a preoperative diagnosis of a hepatic lesion is primarily dependent on imaging studies. Hepatic PEComa may appear as a solitary mass or multiple masses. As Hepatic PEComas have variable histological appearance, these tumors lack specific radiological imaging characteristics. Hepatic PEComa appears as an echogenity on ultrasonography. A more vascular appearance of a mass compared to normal hepatic tissue on Doppler USG favors the diagnosis of PEComa [10]. When Sulphur hexafluoride is administered as the contrast material of advanced ultrasonography, lesions appear hypervascular in the arterial and portal phases [11]. On tomography and MRI PEComas cannot be distinguished from other hepatic masses. Having said that, it is possible to interpret tumors as PEComa, depending on their fat density and vascularity [12]. A computerized tomography reportedly revealed hepatic adenoma and hepatic tumors of mesenchymal origin primarily as the provisional diagnosis due to a smooth contour and marked hypervascularity of the hepatic mass.

The appropriate preoperative diagnostic method for PEComa is still a subject of debate. Fine needle aspiration biopsy (FNAB) is a diagnostic method practiced in many patients. Microscopically, epithelioid, spindle cells and adipocytes can be defined, which makes pathologists consider hepatic PEComa in the differential diagnosis.

They exhibit normochromatic, small nucleotides with round nucleotides [4]. Additionally, they are positively characterized by melanocytic and muscular markers. The most notable immunological markers include HMB-45, Melan A, and SMA [13]. The microscopic examination of our patient revealed diffusely strong staining with HMB45 and SMA as well as an infiltrative growth pattern and nuclear atypia. Furthermore, we excluded other hepatic mass lesions by showing negative markers like cytokeratin, CD117, and AFP.

Because of the rare nature of the disease, there are diagnostic challenges and treatment of hepatic PEComa is debated. A great majorityof the reported hepatic PEComa cases show a benign course although some malignant tumors have also been reported [14]. There are also some cases that show an invasive growth pattern with distant metastasis or recurrence. There is no single standard yet to assess the malignancy grade of a hepatic PEComa. Most of the reported cases underwent surgical resection soon after their diagnosis. This is because most tumors were preoperatively misdiagnosed as HCC or hepatic metastasis. Postoperative complication or recurrence has been rarely reported [15]. We performed open nonanatomic liver resection upon the suspicion of a hepatic adenoma. During surgery, the most important point to pay attention is the surgical margins. Our patient did not suffer any complication during or after the operation.

## 4 Conclusion

Hepatic PEComa is a rare mesenchymal neoplasia and malignant hepatic PEComa has been only rarely reported. The diagnostic approach, treatment modalities, and follow-up procedures are not standard. The exact diagnosis of PEComa is based on histological findings and immunohistochemical properties like HMB-45, SMA, and melan A. Although every neoplasia in the liver cannot be necessarily detected by radiological imaging, various tools including ultrasonography, CT, and MRI can provide important clues for physicians. The main treatment method for the disease is surgical resection with adequate surgical margin. If the tumor size is smaller than 5 cm, and if FNAB result is a benign pathology, it may suffice to follow the patient closely. A longer follow-up period is required to determine the origin, differentiation, and nature of hepatic PEComa.

## Conflicts of interest

None.

## Sources of funding

None.

## Ethical approval

This study was exempt from ethnical approval by our institution.

## Consent

Written consent was obtained from patient for the publication of this case report.

## Author contribution

Mahir Kirnap: Data collection; writing paper.

Gonca Ozgun: Study concept.

Gokhan Moray: Data analysis.

Mehmet Haberal: Data analysis.

## Registration of research studies

Not applicable.

## Guarantor

Mehmet Haberal, MD.
